# Social and environmental risk factors for road traffic injuries among children under five in rural China

**DOI:** 10.1097/MD.0000000000019825

**Published:** 2020-04-24

**Authors:** Yuxi Liu, Meixian Wang, Leni Kang, Chunhua He, Lei Miao, Lingxiao Chen, Siyan Zhong, Jun Zhu, Juan Liang, Qi Li, Yanping Wang, Hanmin Liu

**Affiliations:** a National Office for Maternal and Child Health Surveillance of China; b Key Laboratory of Birth Defects and Related Diseases of Women and Children of the Ministry of Education, West China Second University Hospital, Sichuan University, Chengdu; c Department of Pediatrics, Liupanshui City Maternal and Child Health Care Hospital, Liupanshui; d Department of Pediatrics, Shifang Maternal and Child Health Care Hospital, Shifang, Deyang; e Department of Pediatrics, West China Second University Hospital, Sichuan University, Chengdu, China.

**Keywords:** accidental death, children, primary caregivers, road traffic accidents, rural China

## Abstract

**Background:**

Road traffic injuries (RTIs) have become a considerable issue for children. In China, RTIs are among the top 3 contributors to injury-related mortality and disability-adjusted life years. The present study aimed to evaluate social and environmental factors that may contribute to RTIs among children under 5 in rural areas of China.

**Methods:**

The study was based on 1 year of data (October 1, 2015 to September 30, 2016) from the National Maternal and Child Health Surveillance System (NMCHSS) from all districts in 334 National Maternal and Child Health Surveillance Districts in 30 Chinese provinces, autonomous regions, and municipalities. Data were analyzed to identify environmental, social, and primary caregiver factors related to RTIs among children under 5.

**Results:**

Based on data for the 279 children registered in the NMCHSS during the study period, incidence of RTIs increased with increasing age and was higher for boys than girls. Risk of RTIs depended on distances from the child's home to roads and playgrounds. Enrollment in kindergarten and characteristics of primary caregivers affected risky road behaviors by children. Most primary caregivers (67.4%) reported never using child car seats, and 70.6% reported never using a child helmet. Among primary caregivers without a driver's license, 24.8% reported having driven motor vehicles or motorcycles.

**Conclusions:**

The living environment and behaviors of primary caregivers can affect risk of RTIs in children younger than 5 years in rural China. Road safety awareness should be strengthened at the community and kindergarten levels.

## 1 Introduction

Road traffic injuries (RTIs) cause a significant socioeconomic burden worldwide. Almost 1.35 million people die annually due to RTIs, which cost up to 3% of the gross domestic product in many countries ^[1]^. Over 90% of RTIs occur in low and middle-income countries ^[2,3]^. In addition, approximately 34% of deaths due to RTIs are pedestrians and cyclists ^[4,5]^. Moreover, according to data from the World Health Organization, RTIs are the leading cause of disability for children ^[6]^. In China, RTIs have become the second cause of death among children aged 1 to 14 years, ^[7]^ with a mortality rate of 18.8 per 100,000 in 2013.^[8]^ This is even higher than the rate in Western Pacific countries (17.3 per 100,000) and around the world (17.4 per 100,000). ^[8]^

Children under 5 are involved in RTIs as pedestrians, cyclists, or passengers. Previous work ^[9–11]^ identified several factors contributing to child mortality in RTIs, including factors related to the income and other characteristics of parents or other caregivers; children's characteristics, such as sex, age and the tendency to engage in risky behaviors; and use of safety protection when riding in motorized vehicles or bicycles.

Reduction of RTI-related deaths is included in the third Sustainable Development Goal (Target 3.6). The Chinese government has adopted a series of measures and set specific goals for RTI prevention. The General Office of the State Council of China has implemented the National Disability Prevention Action Plan (2016–2020). ^[12]^ This Plan states that strengthening road traffic management is important for preventing and controlling disability and mortality rates. Additionally, the government has set the target of reducing the death rate caused by RTIs by 6% from 2016 to 2020. ^[12]^ The analysis of RTI risk factors may contribute to prevention efforts.

To provide data for this purpose, the National Office of Maternal and Child Health Surveillance (NMCHSS) conducted a nationwide survey in November 2015. The present study drew on data collected during a 1-year period to evaluate mortality due to RTIs among children under 5 in rural China and identify major risk factors of these incidents. The present results may contribute to the establishment of mechanisms to prevent RTIs in this vulnerable age group.

## 2 Materials and methods

### 2.1 Study subjects

The survey covered all the districts of 334 in 31 provinces, autonomous regions and municipalities of China. This system helps the national and local provincial health management departments supervise and guide health surveillance. Further details about the NMCHSS have been described elsewhere.^[13]^ For the present study, data were extracted on children under 5 years of age who died due to RTIs at one of the 334 surveillance districts between October 1, 2015 and September 30, 2016.

### 2.2 Questionnaire

Data were in the form of responses provided by primary caregivers or other family members to the Road Traffic Accidents Mortality Questionnaire, which was designed by the Chinese National Health Commission and UNICEF to gain information on children under 5 who died due to RTIs. The questionnaire includes 4 parts of questions, including basic information about children, caregivers and families, and the circumstances of the RTIs. All questions are based on relevant risk factors at the scene of accidents, as well as factors related to the primary caregivers and children's living circumstances. Respondents provided informed consent for their anonymized data to be used for research purposes and publication.

### 2.3 Data collection and quality control

A trained investigator filled out the questionnaire during an interview with the respondent. To ensure the quality of the surveyed data, interviews with respondents were conducted within 3 months after the children's death. One worker in each province, prefectural city, and surveillance districts was responsible for the organization, implementation, survey conduction, data review, and results reporting. The researcher was trained to read the survey description and corresponding notes before filling in the questionnaire, and was allowed to call the person in charge at any time if he or she had any questions. At the end of the questionnaire, the same investigator checked its completeness and ensured the data were reliable. The data underwent further quality control at the district, county, prefectural, provincial, and national levels.

### 2.4 Statistical analysis

Results were collected in Microsoft Excel and analyzed using SPSS 22.0 (IBM, Armonk, NY).

## 3 Results

### 3.1 Demographic characteristics of children under 5 who died due to RTIs in rural China

**Figure 1:**
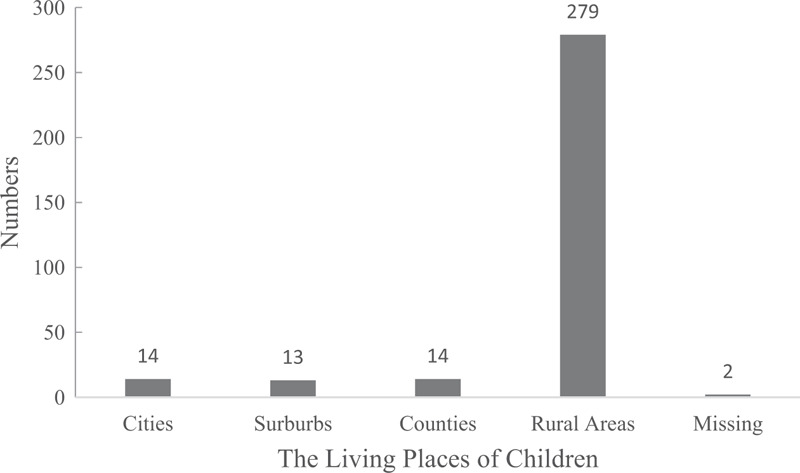
Distribution of the 322 children in the study, by living environment.

In total, data were extracted from the NMCHSS on 322 children who died due to RTIs in China from October 1, 2015 to September 30, 2016, 279 (86.6%) of whom (167 boys) lived in rural areas (Fig. 1, Table 1). The other 43 deaths (13.4%) were excluded. Deaths concentrated mainly among those aged 1 to 4 years, with the highest percentage occurring among those aged 1 year. Nearly 95% of children under 3 years and more than half (55.0%) of children over 3 years were not enrolled in kindergarten (Table 1).

**Table 1:**
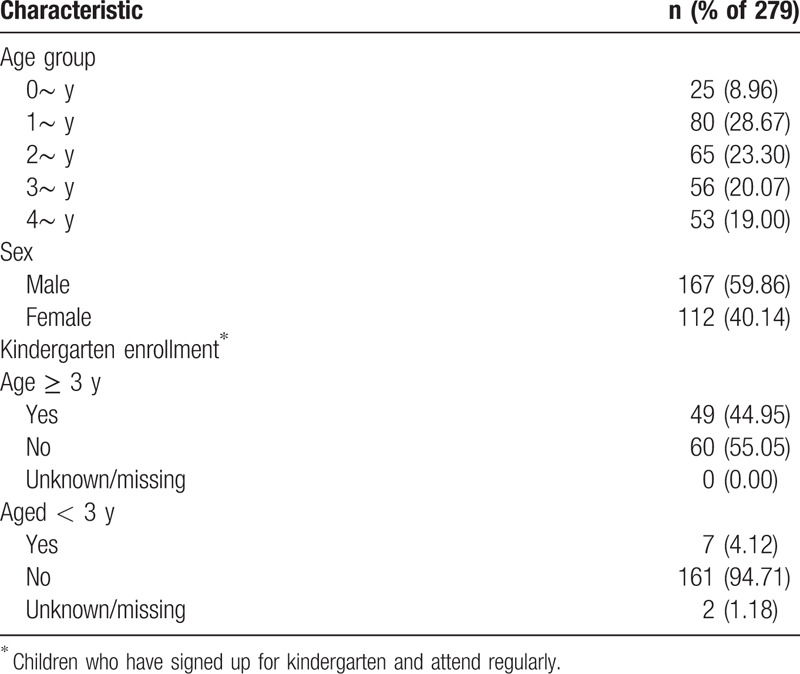
Characteristics of the 279 children in the study.

### 3.2 Environmental risk factors of RTI-related deaths of children under 5 in rural China

**Figure 2:**
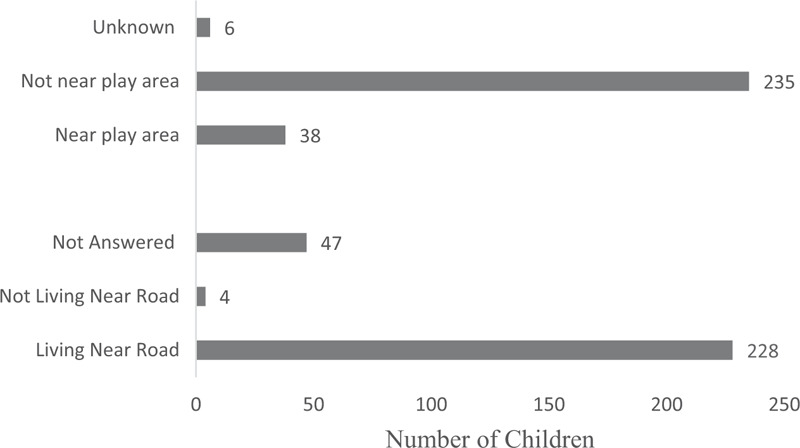
Characteristics of the environment within 100 m of children's homes (n = 279).

The questionnaire collected data on the environment within 100 m of the child's house. Most children (81.72%) lived within 100 m of a road, and most (84.23%) lived more than 100 m from playgrounds or squares appropriate for children's activities (Fig. 2).

### 3.3 Characteristics of primary caregivers of children under 5 who died due to RTIs in rural China

#### 3.3.1 Full-time care and primary caregivers’ relationship to the children

Most children (73.84%) received full-time care from their primary caregivers. The primary caregiver was the mother for over 50% of children, while grandparents took care of 19% of children. Older children were less likely than younger children to receive full-time care from their primary caregivers, and more likely to be cared for by grandparents than by parents (Table 2).

**Table 2:**
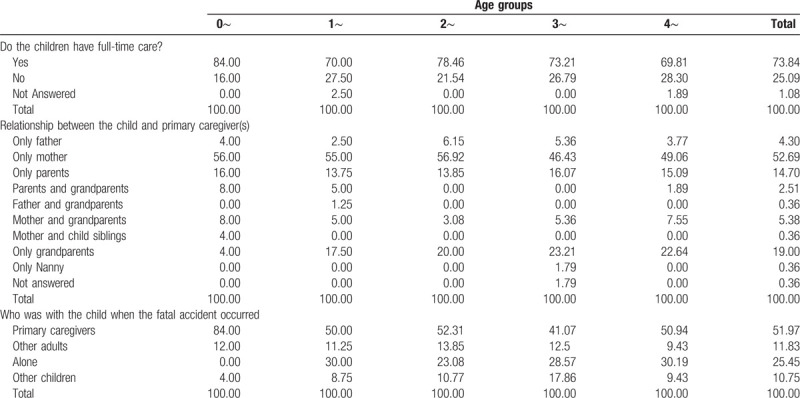
Characteristics of children's primary caregivers.

#### 3.3.2 Children's care situation at the time of RTIs

At the time of road traffic accidents, 51.97% of children were with their primary caregivers at the time of the RTI, while 25.45% of children were alone (Table 2). Among children younger than 1 year, 84% were with their primary caregivers at the time of the RTI. The proportion of children who were alone at the time of the RTI increased as they got older; the proportion of children who were with other children at the time of the RTI was higher at 2 to 3 years of age (Table 2).

#### 3.3.3 Primary caregivers’ driving status

Half (53.41%) of primary caregivers did not have any type of driving license, 20.4% had a motorcycle driving license, and 19.3% had a motor vehicle driving license. Among primary caregivers who had no driving license, 24.83% reported having driven a motorcycle or motor vehicle (Table 3).

**Table 3:**
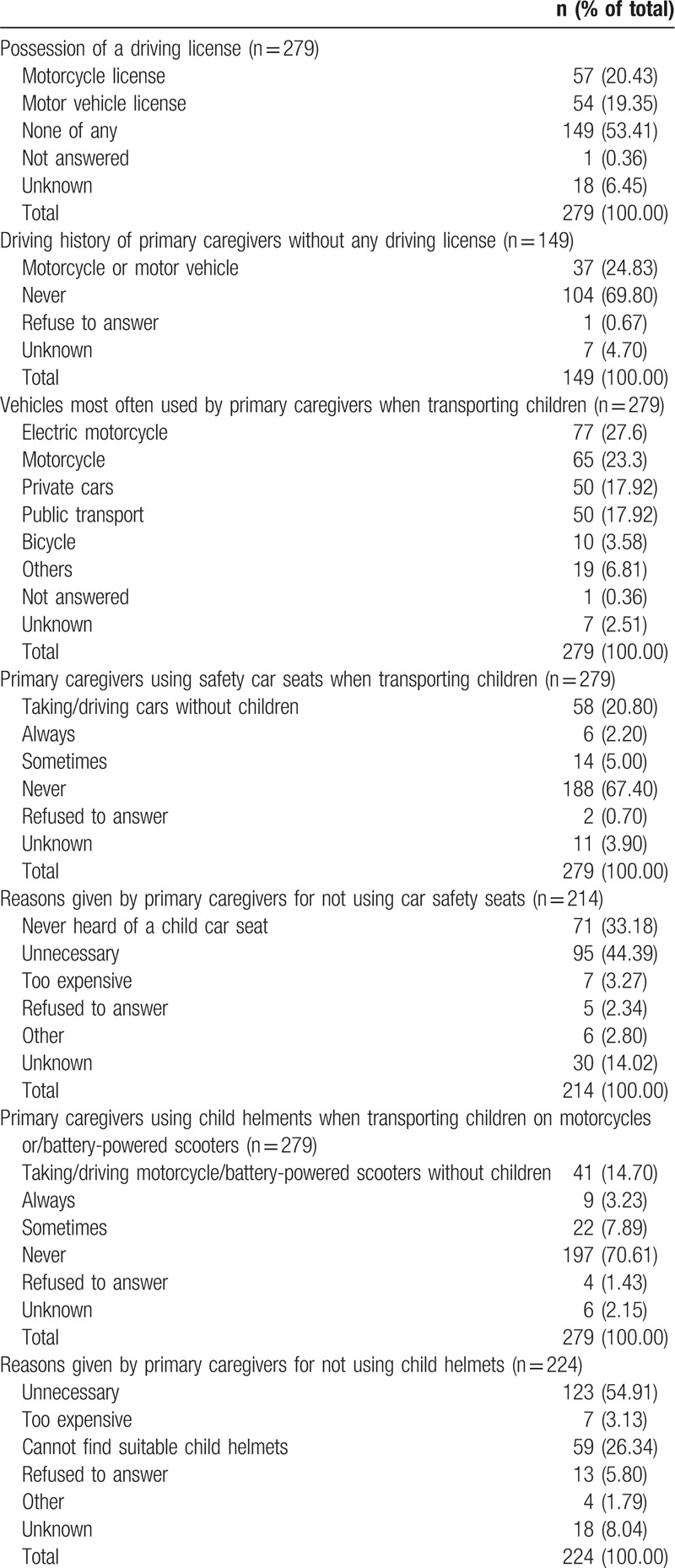
Use of transportation modes and safety equipment by primary caregivers.

More primary caregivers (35.8%) traveled with children by bus or private car, followed by motorcycle (27.6%) and motorbike (23.3%) (Table 3). Two-thirds (67.40%) of primary caregivers who transported children in cars did not use child car safety seats; 44.4% of them believed that such seats were unnecessary, and 33.18% had never heard of them. Over 70% of primary caregivers who transported children on motorcycles did not have a child helmet, more than half thought it was unnecessary, and 26.34% reported being unable to buy suitable child-size helmets (Table 3).

### 3.4 Children's road safety education and risky road behaviors in daily life

#### 3.4.1 Children's road safety education

**Figure 3:**
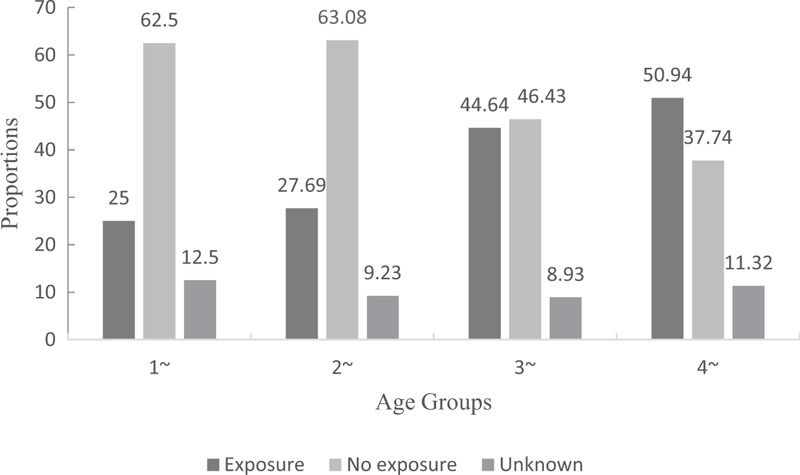
Exposure of children to road safety education from parents, grandparents or kindergartens, stratified by child's age (n = 279).

Among the 254 children from 1 to 4 years old, over half did not have access to any road safety education. More than 60% of children in the “1+” and “2+” age groups did not have access to such education. This proportion decreased with increasing age, which may relate to kindergarten enrollment (Fig. 3).

#### 3.4.2 Children's risky road behaviors in daily life

The questionnaire asked about 5 types of common risky road behaviors: crossing the road, playing on the road, walking on the road, running across the street against a red light, and jaywalking. Among children not enrolled in kindergartens, about 40% of them always or sometimes engaged in risky road behaviors, including walking on roads, jaywalking and playing on roads (Table 4).

**Table 4:**
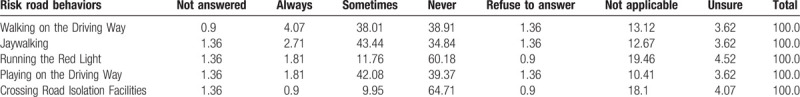
Risky road behaviors among 279 children not enrolled in kindergartens (279, %).

### 3.5 Type of transportation and safety protection at the time of RTIs

**Figure 4:**
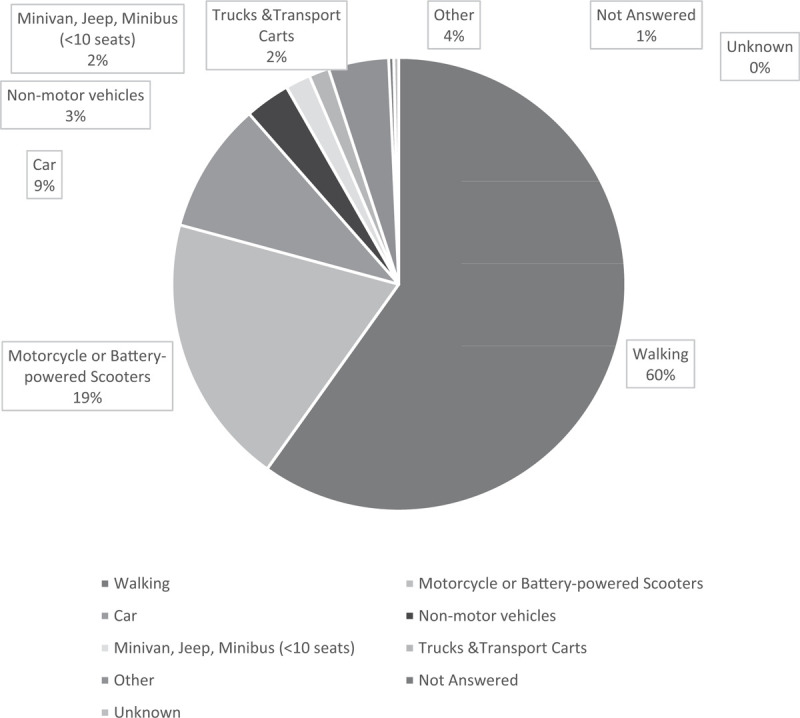
Distribution of types of vehicles used by primary caregivers when transporting children at the time of RTIs (n = 279). RTI = road traffic injuries.

**Figure 5:**
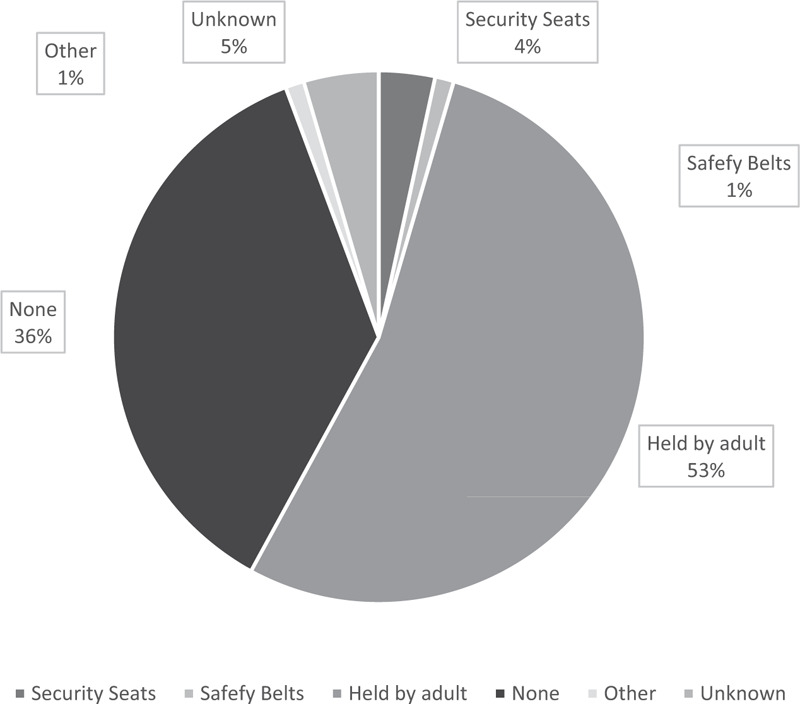
Safety equipment used by primary caregivers to protect children at the time of their RTI (n = 88).

At the time of RTIs, near 60% of children were walking (including being held or carried by an adult) at the time of the RTI, while 20% of them were taking a motorcycle or battery-powered scooters, and 9.32% were riding in a car (Fig. 4). For the 88 of 279 children who were passengers at the time of the RTI, half of children (53.0%) were being held by their primary caregiver, while another 36% of children not have any safety protection (Fig. 5).

## 4 Discussion

This study focused on RTIs among children under 5 in rural China using data obtained from the NMCHSS about the children's characteristics, living environment, and primary caregivers. Our analysis suggests that several external factors influence the risk that young rural children will die in RTIs: age, kindergarten enrollment, use of safety protection, proximity of home to roads, primary caregivers’ road safety awareness, and children's risky behaviors.

The results showed that the percentage of RTIs increased with age. One potential explanation is that children increase their range of activities between 2 and 3 years. One study performed in 2015 in China also found a large number of RTIs among preschool children, which the researchers attributed to children's immature visual system and poor ability to respond to unexpected situations. ^[14]^

Our results suggest that children's risky behaviors are affected by their living environment and exposure to road safety education. Many children who were not enrolled in kindergarten engaged more frequently in risky road behaviors, including walking or playing on roads and jaywalking. Nearly half (46.43%) of children older than 3 years who were not enrolled in kindergarten did not receive road safety education. Compounding this lack of safety education, more than 80% of children in our cohort lived near roads and far from playgrounds. Consistent with our results, previous studies demonstrated that road safety education can modify children's risky road behaviors and reduce the risk of RTIs. ^[15–17]^ Since the most common sources of road safety education for young children are their family members and kindergartens, our results suggest that local communities in rural areas may encourage caregivers to promote enrollment of children in kindergartens, as well as help local kindergartens provide road safety education.

According to data from the NMCHSS, the mortality rate among children under 5 in China decreased 34.5% between 2000 and 2018. ^[18]^ This reduction may be associated with the rapid increase in the number of private cars, motorcycles, and electric motorcycles in rural areas as a result of rapid economic development in China ^[19]^, as well as the fact that by the end of 2018, roads had been paved in 99.6% of towns and 99.5% of incorporated villages. ^[20]^ Nevertheless, the Road Traffic Safety Development Report (2017) indicated that the situation of rural road safety is still deficient, ^[21]^ and that the risk of being involved in RTIs for rural children has increased in recent years.^[22]^

Our data suggest that, at least in rural areas, people's understanding of road safety awareness and related protection measures are not keeping pace with the rapidly changing traffic environment. If primary caregivers engage in risky road behaviors, children may be more likely to do so as well. In our study, 50.9% of primary caregivers often took children on battery-powered scooters or motorcycles, and 19.3% of children were on electric cycles or motorcycles at the time of their RTIs. Of the 86 children who were involved in RTIs as passengers, 79 did not have any specific safety protection (except perhaps being held by an adult). Substantial proportions of primary caregivers had insufficient knowledge about road safety and/or failed to correctly recognize the importance of car seats and child-size helmets. Other research has reported similarly insufficient awareness of road safety in rural areas of China. ^[23]^ Unlike motorcycles, battery-powered scooters require no driving license. Local governments should focus on strengthening the management and control of battery-powered scooters, and encouraging the wearing of helmets by drivers and passengers of motorcycles and battery-powered scooters.

Since many parents in rural China often need to work outside their usual place of residence, even in other provinces, it is likely that they are unable to provide full-time care or to concentrate on child care due to housework or other tasks. The survey showed that the proportion of children with full-time care decreased with increasing children age, which likely influences the risk that the child will be involved in an RTI.

In view of the results of the survey, local government should encourage preschool-age children to attend kindergarten, and it should strengthen safety education, which may help to reduce dangerous road behaviors. Moreover, knowledge and awareness of child protection and road safety should be fostered within the community, which may give caregivers a better understanding of the need for passenger safety equipment. Local governments with sufficient budget resources should improve road safety facilities, such as traffic barriers and fences for pedestrians and cyclists, as well as create appropriate areas for children's activities that reduce the risk of RTIs.

## 5 Limit

Since we found the causes of deaths were similar, such as hemorrhagic shock, therefore, we have not collected the types of lesions in the survey. We will fix this limitation in further studies.

## 6 Conclusions

This cross-sectional survey examined the recent situation of RTIs in children under 5 years of age by monitoring sites in NMCHSS rural areas in China. Occurrence of RTIs was influenced mainly by the limitations of the surrounding environment, lack of comprehensive understanding of road safety and poor monitoring by primary caregivers. To reduce the occurrence of RTIs, local governments should strengthen the awareness of road traffic safety within communities and kindergartens, including the popularization of children's protective equipment.

## Acknowledgments

The authors thank the institutions and staff of the National Maternal and Child Health Surveillance System for data collection.

## Author contributions

**Data analysis:** Yuxi Liu and Meixian Wang.

**Methodology:** Hanmin Liu and Yanping Wang, Leni Kang.

**Data curation:** Jun Zhu, Juan Liang, Lei Miao, Lingxiao Chen, Siyan Zhong.

**Software:** Chunhua He, Leni Kang and Qi Li.

**Writing – original draft:** Yuxi Liu.

**Writing – review & editing:** Yuxi Liu, Meixian Wang, Hanmin Liu and Yanping Wang.
